# Co-infection and co-localization of Kaposi sarcoma-associated herpesvirus and Epstein-Barr virus in HIV-associated Kaposi sarcoma: a case report

**DOI:** 10.3389/fcimb.2023.1270935

**Published:** 2023-10-20

**Authors:** Peter Julius, Guobin Kang, Stepfanie Siyumbwa, Jane Musumali, For Yue Tso, Owen Ngalamika, Trevor Kaile, Fred Maate, Phyllis Moonga, John T. West, Peter Angeletti, Charles Wood

**Affiliations:** ^1^ Department of Pathology and Microbiology, School of Medicine, University of Zambia, Lusaka, Zambia; ^2^ Department of Interdisciplinary Oncology, Louisiana State University Health Sciences Center – New Orleans, New Orleans, LA, United States; ^3^ University Teaching Hospitals, Eye Hospital, Ministry of Health, Lusaka, Zambia; ^4^ Nebraska Center for Virology, University of Nebraska-Lincoln, Lincoln, NE, United States

**Keywords:** Kaposi sarcoma, Kaposi sarcoma-associated herpesvirus, Epstein-Barr virus, co-infection, co-localization, HIV, Zambia

## Abstract

Kaposi sarcoma (KS), a multifocal vascular neoplasm frequently observed in HIV-positive individuals, primarily affects the skin, mucous membranes, visceral organs, and lymph nodes. KS is associated primarily with Kaposi sarcoma-associated herpesvirus (KSHV) infection. In this case report, we present a rare occurrence of co-infection and co-localization of KSHV and Epstein-Barr virus (EBV) in KS arising from the conjunctiva, which, to our knowledge, has not been reported previously. Immunohistochemistry (IHC), DNA polymerase chain reaction (PCR), and EBV-encoded RNA *in situ* hybridization (EBER-ISH) were utilized to demonstrate the presence of KSHV and EBV infection in the ocular KS lesion. Nearly all KSHV-positive cells displayed co-infection with EBV. In addition, the KS lesion revealed co-localization of KSHV Latency-Associated Nuclear Antigen (LANA) and EBV Epstein Barr virus Nuclear Antigen-1 (EBNA1) by multi-colored immunofluorescence staining with different anti-EBNA1 antibodies, indicating the possibility of interactions between these two gamma herpesviruses within the same lesion. Additional study is needed to determine whether EBV co-infection in KS is a common or an opportunistic event that might contribute to KS development and progression.

## 1 Introduction

Kaposi Sarcoma (KS) is a multifocal angio-proliferative tumor commonly seen in individuals with HIV (Vangipuram and Tyring, 2019) and occasionally in individuals without HIV (Kumar et al., 1989; Seleit et al., 2011; Kodra et al., 2015) infection. The causative agent of KS is a human γ-herpesvirus, the Kaposi sarcoma-associated herpesvirus (KSHV), also known as human herpesvirus 8 (Vangipuram and Tyring, 2019). While co-infection with Epstein-Barr virus (EBV), another potentially oncogenic γ-herpesvirus, has been identified in various malignancies (Mui et al., 2017; Bigi et al., 2018; Ghoreshi et al., 2023); its occurrence and significance in KS have not been studied. This report presents a case of ocular KS with co-infection and co-localization of KSHV and EBV within the tumor tissue.

During our previous investigation, exploring the potential associations between oncogenic viruses and ocular surface squamous neoplasia (OSSN) (Julius et al., 2021; Julius et al., 2022; Julius et al., 2023), we encountered a patient with ocular KS among our cohort of 458 patients recruited with ocular surface tumors at the University Teaching Hospitals (UTH) Eye Hospital in Lusaka, Zambia. As an incidental finding, the ocular surface KS tumor analysis revealed the co-infection and co-localization of KSHV and EBV. The presence of EBV in this ocular KS tissue was confirmed by immunohistochemistry (IHC) with two different antibodies targeting EBV nuclear antigen 1 (EBNA1), by DNA polymerase chain reaction (PCR) for EBNA1, and by EBV-encoded RNA *in situ* hybridization (EBER-ISH).

## 2 Case presentation

**Figure 1:**
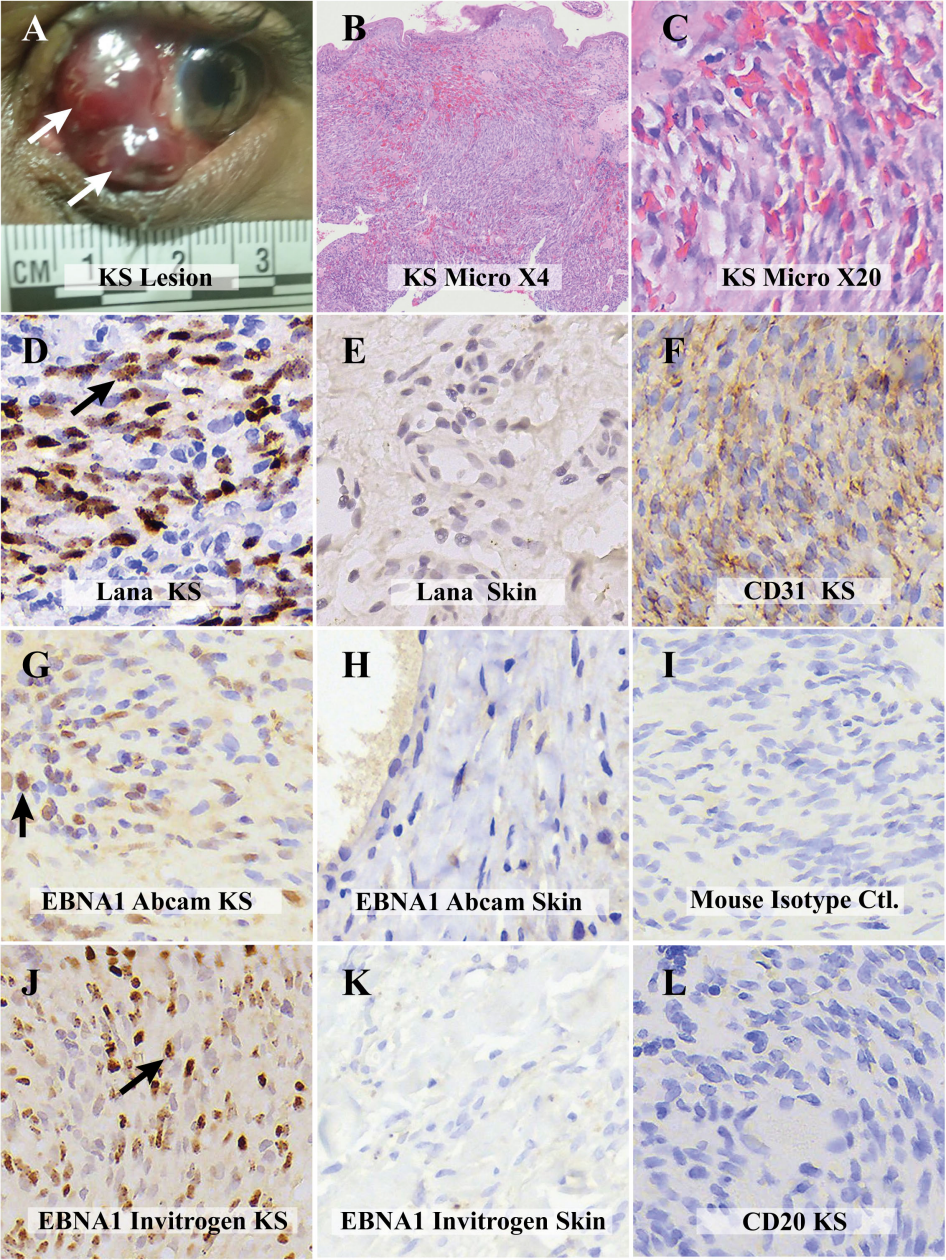
Comparative visualization of Kaposi Sarcoma (KS) from the Ocular Surface and Control Samples: **(A)** Macroscopic view of the ocular surface affected by KS. **(B)** Histological examination at low magnification (X4) of the KS lesion, stained with hematoxylin and eosin. **(C)** Detailed histological view at higher magnification (X20) derived from Image panel **(B)**. **(D)** Immunohistochemical (IHC) analysis. LANA positivity in the ocular KS lesion, utilizing Abcam’s Rat anti-LANA primary antibody. **(E)** IHC control for LANA with a KS-negative skin biopsy. **(F)** Magnified view emphasizing endothelial cell markers CD31 with positive IHC staining in the ocular KS lesion. **(G)** IHC depiction revealing EBNA1 positivity in the KS lesion, employing Abcam’s mouse anti-EBNA1 primary antibody. The image exhibits both diffuse and punctate nuclear staining patterns.**(H)** IHC analysis using Abcam’s mouse anti-EBNA1 primary antibody, indicating EBNA1 absence in a skin biopsy without KS. **(I)** Mouse isotype control for IHC. **(J)** IHC representation highlighting EBNA1 positivity in the ocular KS lesion, using Invitrogen’s mouse anti-EBNA1 primary antibody. The staining presents a punctate nuclear pattern. **(K)** IHC examination employing Invitrogen’s mouse anti-EBNA1 primary antibody, demonstrating the absence of EBNA1 in a skin biopsy without KS. **(L)** IHC analysis with the CD20 antibody, confirming the lack of B cells in the KS lesion. All digital microscopic images of the stained slides were captured using the MoticEasyScan Pro 6 scanner (Motic, USA) and analyzed using the Motic DSAssistant VM 3.0 software. For the journal presentation, images were cropped to a 500x500 pixel resolution using Microsoft’s Paint 3D software.

We report the case of a 43-year-old male who was diagnosed with HIV infection 10 months before seeking care at the University Teaching Hospital’s Eye Hospital in Lusaka, Zambia. The patient had a previous diagnosis of allergic conjunctivitis and presented with acute-onset symptoms in his left eye. The patient reported experiencing swelling, redness, pain, itchiness, excessive tearing, foreign body sensation, reduced vision, and growth on the surface of the left eye. The duration of these symptoms was approximately two months before the patient decided to seek medical attention. The patient had reported a history of working as a welder with occasions of not using face protective gear. He was prescribed Dexamethasone eye drops on his local clinic visit. Upon clinical examination, a red fleshy lesion was observed, primarily located at the nasal limbus, involving the bulbar conjunctiva, caruncle, cornea, and upper and lower fornix (**Figure 1A**). There were no signs of inflammation. The patient’s visual acuity was compromised at 6/12 (This means that while a person with normal vision can discern an object clearly from 12 meters away, our patient required 6 meters to attain the same clarity). Following the clinical assessment, an impression of OSSN was initially considered with a differential diagnosis of KS.

The conjunctival tumor was surgically excised and subsequently divided into two parts for different analysis. One portion was fixed in 10% neutral buffered formalin for Formalin-fixed paraffin-embedded (FFPE) tissue blocks. Simultaneously, the other segment was placed in RNAlater solution for 24 hours to preserve the nucleic acids and then frozen at -80°C for further molecular investigations. Two independent histopathologists, PJ and FM, confirmed the diagnosis of conjunctiva KS after analyzing hematoxylin and eosin (H&E) FFPE-stained tissue sections (**Figures 1B, C**). The lesion section was examined with immunohistochemistry (IHC) targeting Kaposi sarcoma-associated herpesvirus- encoded latency-associated nuclear antigen (LANA) protein using an anti-LANA Rat monoclonal antibody LN53 (Ab4103, Abcam, USA) at a 1/200 dilution (**Figure 1D**), to confirm the KS diagnosis. The specificity of the LANA antibodies was verified using the BC3 cell line as a positive control (**Supplement Figures 2**) and KSHV-negative skin tissue (**Figure 1E**). Since endothelial cells are the targeted cells infected by KSHV, IHC against endothelial cell markers CD-31 (**Figure 1F**) was performed and shown to be positive in this KS tissue. Since B lymphocytes are the major cell type targeted for EBV infection, the ocular KS tissue was stained for human CD20 (anti-Human CD20cy, mouse monoclonal antibody [L26] (M0755, Dako, USA) at a 1/200 dilution) by IHC. No B lymphocytes were observed among the intratumoral infiltrating leucocytes (**Figure 1L**).

**Figure 2:**
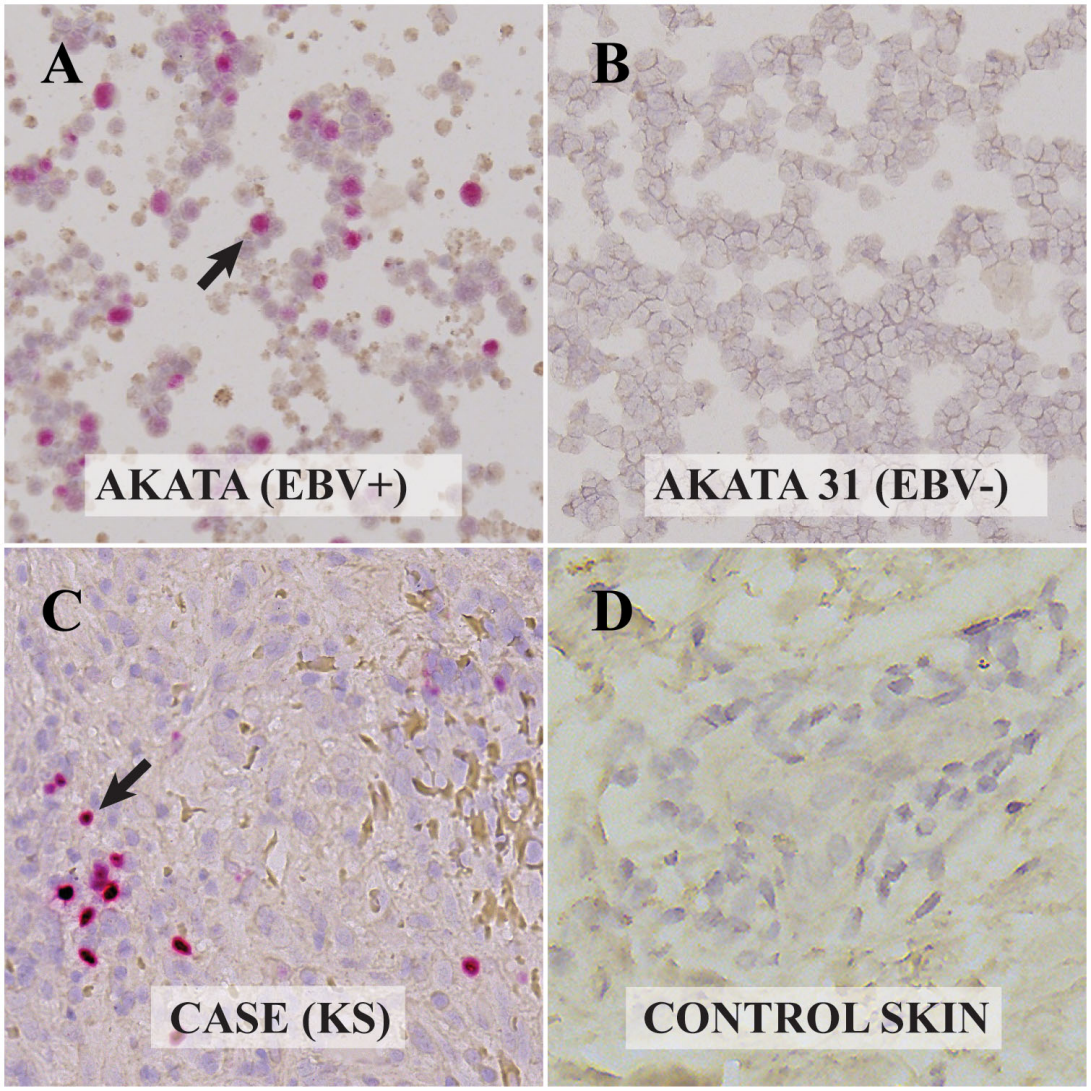
Visualization of Epstein Barr Virus (EBV)-encoded RNAs in control and Kaposi Sarcoma (KS) cells via RNAscope *In-Situ* Hybridization (ISH): **(A)** Akata cell line infected with EBV. The presence of EBV-encoded small RNAs (EBER-RNAscope) is evident from the red dots, highlighted by the arrow. **(B)** EBV-negative Akata 31 cell line. The absence of EBER with RNAscope is observed, indicating no EBV presence. **(C)** KS lesion from the ocular surface. Red dots and the arrow point to the EBER RNAscope positive cell, confirming the presence of EBV. **(D)** Skin biopsy without KS. The absence of red dots indicates EBER-RNAscope negativity, indicating no EBV presence. All digital microscopic images of the stained slides were captured using the MoticEasyScan Pro 6 scanner (Motic, USA) and analyzed using the Motic DSAssistant VM 3.0 software. For the journal presentation, images were cropped to a 500x500 pixel resolution using Microsoft’s Paint 3D software.

Since we have previously shown that there is a high prevalence of EBV infection of the OSSN (Julius et al., 2022), we then tested the tumor tissues for the presence of EBV by IHC on the FFPE ocular KS tumor tissue sections to assess the expression of latent Epstein–Barr virus nuclear antigen 1 (EBNA1) and CD20 to identify the presence of EBV protein expression in the tumor microenvironment and the presence of B cells in the lesion, respectively. Two primary antibodies targeting EBNA1 were used for IHC analysis to confirm the presence of EBV coinfection. Anti-EBV nuclear antigen/EBNA1 mouse monoclonal antibody E1-2.5 (ab8329, Abcam, USA) was used at 1/1000 dilution, and anti-EBV nuclear antigen/EBNA1 mouse monoclonal antibody EBS-I-024 (MA5-33321, Invitrogen, USA) was used at 1/200 dilution. The IHC procedure followed our previously reported protocol (Julius et al., 2022). In brief, the tissue slices were incubated with the primary antibodies after deparaffinization, rehydration, endogenous peroxidase activity blocking, and antigen retrieval, then treated with anti-mouse horse radish peroxidase (HRP)-labeled secondary antibody (K4001, Dako, USA). Our results demonstrated the presence of EBV infection in the tumor cells using both anti-EBV nuclear antigen/EBNA1 antibodies from Abcam and Invitrogen (**Figures 1G, J**, respectively). The mouse isotype control showed no EBNA1 staining (**Figure 1I**). Positive controls utilized the Akata cell line (EBV positive) were positive for EBNA1 using the anti-EBNA1 antibodies from both Abcam (**Supplement Figure 1A**) and Invitrogen (**Supplement Figure 1C**). In contrast, both anti-EBNA1 antibodies from Abcam and Invitrogen were tested negative in the normal non-KS skin tissue (**Figures 1H, K**, respectively) and negative control BC3 (EBV negative) cell line (**Supplement Figures 1B, D**). The presence of EBV in the ocular KS tissue was further confirmed with RNAscope ISH (**Figure 2C**) to detect the presence of EBV-encoded small RNAs (EBER) (EBER1 probe, 310271, ACDbio, USA) according to the manufacturer’s protocol (**Figure 2**). Specificity of the EBER RNAscope ISH was demonstrated with positive signal only in the Akata cell line (EBV positive) (**Figure 2A**), and no signals were detected in the control AKATA31 (EBV negative) cell line (**Figure 2B**) and the EBV negative skin tissue (**Figure 2D**).

**Figure 3:**
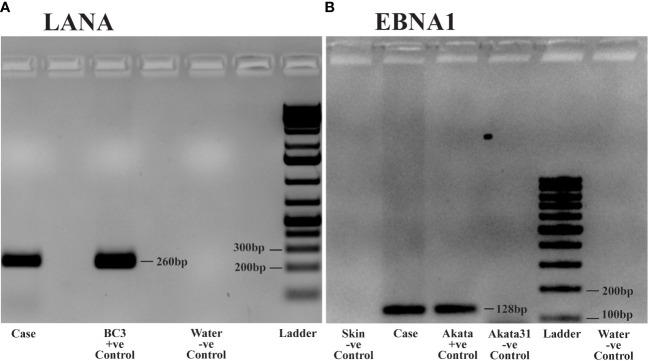
DNA Polymerase Chain Reaction (PCR) analysis of Kaposi Sarcoma (KS) tumor from the ocular surface: DNA was extracted from frozen tissue samples of the KS tumor for PCR analysis. **(A)** PCR detecting Kaposi sarcoma-associated herpesvirus (KSHV) using Latency-Associated Nuclear Antigen (LANA) primers. Case: Patient sample demonstrating KSHV positivity. Positive Control: BC3 cell line, known to be infected solely by KSHV. Negative Control: Water. **(B)** PCR detecting Epstein Barr virus (EBV) using Epstein Barr virus Nuclear Antigen-1 (EBNA1) primers. Case: Patient sample showing EBV positivity. Positive Control: Akata cell line, known to be infected solely by EBV. Negative Controls: Akata31 cell line, normal skin, and water.

To further confirm the presence of KSHV and EBV infection in the tumor tissues, PCR was performed using DNA extracted from frozen ocular KS tumor tissue with the Qiagen DNeasy Blood and Tissue kit (Qiagen Inc., Valencia, CA, USA). Human beta-globin was PCR amplified from all samples to confirm high-quality DNA. KSHV LANA PCR was carried out using LANA-specific primers, forward: 5’- TGGATCTCGTCTTCCATCCTTTCCC -3’, reverse 5’- GCCAGTAGCCCACCAGGAG -3’. The PCR conditions were set as follows: Initial incubation at 95°C for 4 minutes, 40 cycles comprising denaturation at 95°C for 30 seconds, annealing at 62°C for 30 seconds, extension at 72°C for 15 seconds, and the final extension step at 72°C for 1 minute. This produced an amplicon size of 260 bp (**Figure 3A**). The BC3 cell line (KSHV positive) was employed as a positive control. For the detection of EBV, EBNA1 PCR was carried out using EBNA-1 specific primers, forward: 5’- TTTGCTGAGGGTTTGAAGGATGCG -3’, reverse: 5’- ATAGGTGGAAACCAGGGAGGCAAA -3’. The PCR conditions were identical to LANA, except the annealing temperature was at 67°C. This produced an amplicon of 128 bp. The Akata (EBV positive) and the Akata-31 (EBV negative) cell lines were employed as positive and negative controls, respectively. Other negative controls included skin tissue without KS and water (**Figure 3B**). Our PCR results confirmed the presence of both KSHV and EBV viral DNA in this ocular KS tissue, as shown in **Figures 3A, B**, respectively.

**Figure 4:**
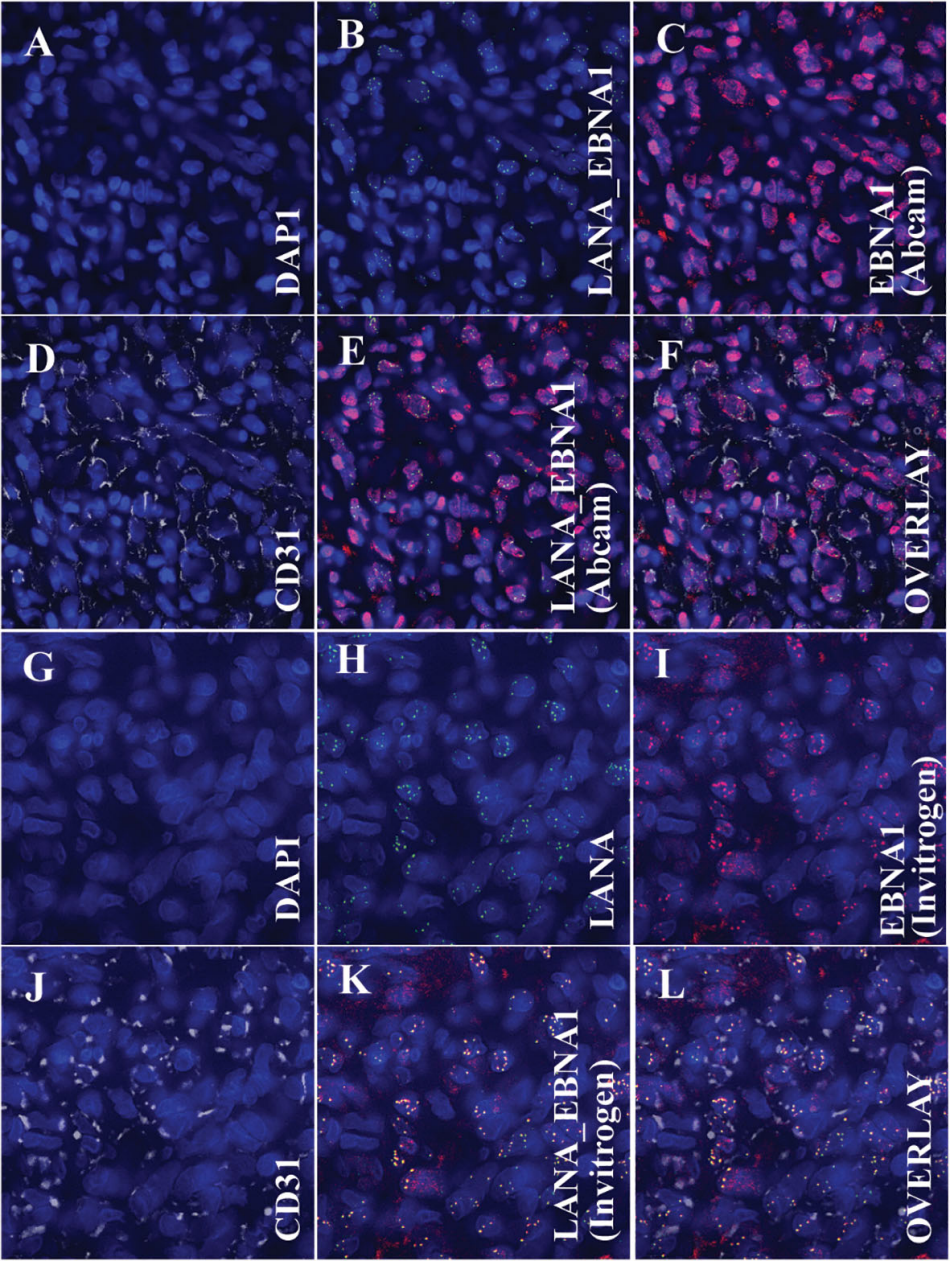
Immunofluorescence Assay (IFA) showing the colocalization of the Kaposi sarcoma-associated herpesvirus-encoded latency-associated nuclear antigen (LANA) protein and Epstein Barr virus’s nuclear antigen 1 (EBNA1) with endothelial cell markers, specifically CD31. **(A–F)** (Using EBNA1 from Abcam): **(A)** Nuclear staining with DAPI. **(B)** Overlay image of DAPI and LANA, denoting the presence of KSHV within the cell nucleus. **(C)** Overlay image of DAPI and EBNA1 (Abcam antibody), indicating the presence of EBV within the cell nucleus. **(D)** Overlay image of DAPI and CD31, emphasizing that the tumor cells exhibit endothelial cell markers. **(E)** Tri-overlay of A, B, and C, illustrating the colocalization of EBNA1 and LANA within tumor cells. **(F)** Quad-overlay of A, B, C, and D, highlighting the colocalization of EBNA1 and LANA in CD31-positive tumor cells. **(G–L)** (Using EBNA1 from Invitrogen): **(G)** Nuclear staining with DAPI. **(H)** Overlay image of DAPI and LANA, denoting the presence of KSHV within the cell nucleus. **(I)** Overlay image of DAPI and EBNA1 (Invitrogen antibody), indicating the presence of EBV within the cell nucleus. **(J)** Overlay image of DAPI and CD31, emphasizing that the tumor cells exhibit endothelial cell markers. **(K)** Tri-overlay of G, H, and I, illustrating the colocalization of EBNA1 and LANA within tumor cells. **(L)** Quad-overlay of G, H, I, and J, highlighting the colocalization of EBNA1 and LANA in CD31-positive tumor cells. All images were captured at 40X magnification with a KEYENCE microscope and analyzed using the KEYENCE BZ-X800 Analyzer. For journal presentation, images were cropped to a 300x300 pixel resolution using Microsoft’s Paint 3D software.

To determine whether the ocular KS tumor cells are co-infected by both KSHV and EBV (**Figure 4**), multi-colored immunofluorescence assays (IFA) were used to detect the co-localization of both KSHV LANA and EBV EBNA1 proteins in the tumor cells according to previously published methodology (Wang et al., 2014). IFA for EBV was carried out using anti-EBNA1 mouse monoclonal antibodies from Abcam and Invitrogen as primary antibodies, and anti-LANA Rat monoclonal antibody LN53 was used as a primary antibody against KSHV. Alexa Fluor donkey anti-mouse IgG and anti-rat IgG from Invitrogen were used as secondary antibodies. As shown in **Figures 4C, I** (Abcam and Invitrogen, respectively), the anti-EBNA1 antibody indicated the presence of EBNA1 proteins (red signals) within the nucleus of the tumor cells, where LANA (**Figures 4B, H**) proteins (green signals) were also detected. Similarly, the Abcam and Invitrogen anti-EBNA1 antibodies also detected the presence of EBNA1 proteins within the nucleus of LANA-positive cells (**Figures 4E, K**, respectively). Notably, the EBNA1 antibody from Abcam displayed two clear staining patterns in both IHC (**Figure 1G**) and IFA (**Figure 4C**): a diffuse signal and punctate nuclear staining (Abcam, 2023). The EBNA1 antibodies from Invitrogen showed a more distinct punctate staining in both IHC (**Figure 1J**) and IFA (**Figure 4I**). These findings detected the simultaneous existence and interplay of EBV/EBNA1 and KSHV/LANA in the KS tumor derived from the conjunctiva for our patient.

Since our human CD20 IHC staining showed a lack of B lymphocytes within the ocular KS tissue, we stained the tumor tissue against human endothelial cell markers CD31 (**Figures 4D, J**), using IFA to identify the cell type co-infected with EBV and KSHV. The primary antibodies used in this assay were anti-LANA rat monoclonal antibody LN53, anti-EBNA1 mouse monoclonal from either Abcam or Invitrogen and anti-CD-31 (white signals) rabbit monoclonal antibody (EPR3094, ab76533, Abcam, USA, at a 1/200 dilution). Co-staining of CD31 with either Abcam anti-EBNA1 (**Figure 4F**) or Invitrogen (**Supplement Figure 4L**) indicated that the EBV-infected cells are also endothelial cells, same as the KSHV-infected cell type as demonstrated by the co-staining of CD31 with LANA (**Figures 4F, L**).

To ensure the observed IHC and IFA signals were not a result of antibody cross-reactivity, we conducted staining experiments on the Akata (EBV positive, KSHV negative) and Akata31 (EBV negative, KSHV negative) cell lines using anti-KSHV LANA antibodies. Our findings revealed no cross-reactivity (**Supplementary Figures 1F**, **3B, E**). Similarly, when the BC3 (EBV negative, KSHV positive) cell line was stained with anti-EBNA1 antibodies from both Abcam (**Supplementary Figures 1B**, **3H**) and Invitrogen (**Supplementary Figures 1D**, **3K**), no cross-reactivity was observed. To further validate the absence of “bleed through” signals during multi-colored IFA, we stained slides exclusively with either anti-EBNA1 alone (**Supplementary Figures 4B, F**) or anti-LANA alone (**Supplementary Figures 4J**), followed by scanning the slide at channels used for both EBNA1 (AF647, red color) and LANA (AF488, green color) (**Supplementary Figures 4C, G, K**) so that both color channels were displayed separately to verify that signals from one color were not bleeding into the other channel. Our results conclusively demonstrated that the signals were genuine and not a result of signal bleed-through.

## 3 Discussion

This case report presents an incidental yet significant finding of co-infection and co-localization of KSHV and EBV in a KS tumor from the conjunctiva in an HIV-positive patient from Zambia. This sheds light on potential co-infections in KS and the implications of viral interactions. KSHV and EBV are potentially oncogenic viruses commonly found in individuals with HIV infection, and their co-infection has been implicated in primary effusion lymphoma. EBV infection is linked to Hodgkin’s and non-Hodgkin’s lymphoma, nasopharyngeal carcinoma, and gastric carcinoma (Mui et al., 2017; Bigi et al., 2018; Ghoreshi et al., 2023), whereas KSHV infection is predominantly associated with KS, primary effusion lymphoma, and Castleman’s disease (Bigi et al., 2018; Sánchez-Ponce and Fuentes-Pananá, 2021). While both KSHV and EBV have been reported to coexist in primary effusion lymphoma (PEL) cells, Kaposi sarcoma (KS) has traditionally been associated solely with KSHV. Thus, a co-infection of KS with both EBV and KSHV within the same cells would represent a novel finding of significant importance. To our knowledge, this is the first documentation of EBV and KSHV co-infection in KS tumor cells using various methods.

Previously, Henghold et al. (1997) reported KSHV and EBV detection in iatrogenic KS using DNA PCR and Southern Blot on fresh frozen tissue. However, they did not demonstrate EBV and KSHV co-localization in the KS tumor cells. EBV and KSHV co-infection has also been reported in odontogenic lesions using PCR but without immunohistology confirmation (Alsaegh et al., 2023). While previous studies have established the infection of endothelial cells by EBV (Jones et al., 1995), the association between EBV infection and KS has not been reported. Our findings of the simultaneous presence of KSHV and EBV in KS tissues are intriguing. It suggests that an unknown interaction between the two viruses might play a role in the pathogenesis of certain KS types. It is crucial to understand the mechanisms underlying their interaction and their impact on KS development and progression. However, as this is a case report, additional study is needed to determine whether EBV co-infection in KS is a common or an opportunistic event that might contribute to KS development and progression.

The EBNA1 antibody from Abcam exhibited both diffuse and punctate signals. However, a predominantly punctate pattern was observed in IFA with the Invitrogen EBNA1 antibody. The variation in EBNA1 IHC staining observed between the Abcam and Invitrogen antibodies may stem from the distinct epitopes chosen by each company to produce their respective mouse monoclonal antibodies. Nevertheless, our results support the dual staining pattern observed with IHC for both EBNA1 antibodies. Interestingly, co-localization of both LANA and EBNA-1 can be observed in the same nucleus. The implications of their co-localization should be further investigated. Given that EBV and KSHV are closely related double-stranded DNA gamma herpesviruses with potential sequence similarities, it was imperative to ensure no antibody cross-reactivity. To confirm the specificity of our antibodies, we tested them on positive and negative cell lines, as well as normal skin tissue, using the EBV/EBNA1 and KSHV/LANA markers. No cross-reactivity was detected, bolstering the credibility of our findings. Our use of both positive and negative controls further validated the results. Additionally, we conducted experiments to rule out any signal “bleed through” between LANA and EBNA1 signals. By staining for each separately, we confirmed no interference between the two signals when viewed individually.

Because co-infection with EBV and KSHV in primary effusion lymphoma was reported to be associated with a more aggressive disease course and poor prognosis (McHugh et al., 2017; Bigi et al., 2018; Gruffat and Manet, 2018), it would be relevant to know the clinical implication of coinfection in KS. We could not establish a meaningful clinical pathologic correlation due to the lack of patient clinical outcome data. Future research on KSHV and EBV coinfection in KS should collect and analyze pertinent clinical data, such as patient demographics, symptoms, disease progression, treatment history, and outcomes, to facilitate a complete interpretation of EBV and KSHV co-infection findings.

In conclusion, we report a case that showed co-infection and co-localization of KSHV and EBV in KS from the conjunctiva using PCR, IHC, IFA, and ISH. This provides valuable insights into potential co-infection in KS and the role of viral interactions.

## Data availability statement

The raw data supporting the conclusions of this article will be made available by the authors, without undue reservation.

## Ethics statement

The University of Zambia Biomedical Research Ethics Committee (IRB # 015-05-17), the Zambia National Health Research Authority, the University of Nebraska-Institutional Lincoln Review Board (IRB # 20170817442FB), and the Institutional Review Board at Louisiana State University (IRB # 2252) reviewed and approved the research. The studies were conducted in accordance with the local legislation and institutional requirements. The participant provided their written informed consent to participate in this study. Written informed consent was obtained from the participant/patient(s) for the publication of this case report.

## Author contributions

PJ: Conceptualization, Writing – review & editing, Data curation, Formal Analysis, Investigation, Methodology, Visualization, Writing – original draft. GK: Data curation, Investigation, Visualization, Writing – review & editing, Validation. SS: Data curation, Visualization, Writing – review & editing, Formal Analysis, Software. JM: Visualization, Writing – review & editing, Investigation. FT: Investigation, Visualization, Writing – review & editing, Validation. ON: Validation, Writing – review & editing, Resources. TK: Validation, Writing – review & editing, Investigation, Supervision. FM: Investigation, Writing – review & editing, Visualization. PM: Investigation, Writing – review & editing, Resources. JW: Resources, Writing – review & editing, Supervision, Validation. PA: Resources, Supervision, Validation, Writing – review & editing, Conceptualization, Formal Analysis, Funding acquisition, Methodology. CW: Conceptualization, Funding acquisition, Resources, Writing – review & editing.

## Supplementary material

Supplementary Figure 1 Demonstrating Antibody Specificity through Immunohistochemistry (IHC). Given the potential sequence similarities between EBV and KSHV, both double-stranded DNA gamma herpesviruses, we conducted tests on Akata, Akata31, and BC3 cell lines to ensure no cross-reactivity of our antibodies. Image A: Akata Cell Line (EBV Infected): This image shows the cells stained with Epstein Barr virus’s nuclear antigen 1 (EBNA1) from Abcam. The positive staining confirms the presence of EBV infection. Image B: BC3 Cell Line: The cells were stained with EBNA1 from Abcam. The negative staining result confirms no cross-reactivity of EBNA1 with KSHV-infected BC3 cells. Image C: Akata Cell Line (EBV Infected): This image shows the cells stained with EBNA1 from Invitrogen. The positive staining indicates EBV infection. Image D: BC3 Cell Line: The cells were stained with EBNA1 from Invitrogen. The negative result confirms the absence of EBNA1 cross-reactivity with KSHV-infected BC3 cells. Image E: BC3 Cell Line (KSHV Infected): This image shows the cells stained with Kaposi sarcoma-associated herpesvirus-encoded latency-associated nuclear antigen (LANA) protein. The positive staining confirms KSHV infection. Image F: Akata Cell Line (EBV Infected): The cells in this image were stained with LANA. The negative staining result confirms no cross-reactivity of LANA with EBV-infected Akata cells. Note: All digital microscopic images of the stained slides were captured at X80 magnification using the MoticEasyScan Pro 6 scanner (Motic, USA) and analyzed using the Motic DSAssistant VM 3.0 software. For the journal presentation, images were cropped to a 300x300 pixel resolution using Microsoft’s Paint 3D software.

Supplementary Figure 2 Representative Immunofluorescence Assay Images of BC3 Cell Lines at 40X Objective Lens: BC3 cell lines serve as positive controls for LANA staining to detect Kaposi Sarcoma Herpes Virus (KSHV) infection. Image A: DAPI stain highlighting the nuclear boundary of BC3 cells. Image B: Detection of KSHV using the LANA Rat monoclonal antibody [LN53] (Ab4103). Image C: Overlay of DAPI and LANA (from Images A & B), confirming KSHV presence in the cell nucleus. Note: All digital images were captured at 40X magnification with a KEYENCE microscope and analyzed using the KEYENCE BZ-X800 Analyzer. For journal presentation, images were cropped to a 300x300 pixel resolution using Microsoft’s Paint 3D software.

Supplementary Figure 3 Representative Immunofluorescence Assay Images using 40X Objective Lens: Demonstrating Antibody Specificity. Akata Cell Line (EBV Infected) images A-D). Image A: DAPI staining. Image B: Negative staining using Abcam’s Rat anti-LANA antibody, confirming LANA’s absence of cross-reactivity with EBV-infected cells. Image C: Overlay of A and B, emphasizing no cross-reactivity between LANA and EBV. Akata31 Cell Line (Non-EBV Infected) images D-F. Image D: DAPI staining. Image E: Negative staining using Abcam’s primary Rat anti-LANA, confirming LANA’s absence of cross-reactivity with Akata31. Image F: Overlay of D and E, emphasizing no cross-reactivity between LANA and the non-EBV-infected cell line. BC3 Cell Line (KSHV Infected) images G-I): Image G: DAPI staining highlighting the nuclear boundary. Image H: Negative staining using the EBNA1 antibody from Abcam, confirming EBNA1’s absence of cross-reactivity with KSHV-infected cells. Image I: Overlay of G and H, emphasizing no cross-reactivity between ABCAM’s EBNA1 and KSHV LANA. Image J: DAPI. Image K: Negative staining using the EBNA1 antibody from Invitrogen, confirming EBNA1’s absence of cross-reactivity with KSHV-infected cells. Image L: Overlay of J and K, emphasizing no cross-reactivity between Invitrogen’s EBNA1 and KSHV LANA. Note: All images were captured at 40X magnification with a KEYENCE microscope and analyzed using the KEYENCE BZ-X800 Analyzer. For journal presentation, images were cropped to a 300x300 pixel.

Supplementary Figure 4 Representative Immunofluorescence Assay Images at 40X Objective Lens: Ensuring No Signal Bleeding Across Color Channels. To ensure no “bleed through” signal from LANA and EBNA1 potentially confounding results, we stained slides exclusively with either anti-EBNA1 alone or anti-LANA alone, followed by scanning the slide at channels used for both EBNA1 (AF647, red color) and LANA (AF488, green color). Both color channels were displayed separately to verify that signals from one color were not bleeding into the other channel. EBV Detection Using Abcam’s EBNA1 Antibody: Image A: DAPI staining highlighting the nuclear boundary of the Kaposi sarcoma (KS) cells. Image B: EBNA1 antibody from Abcam at channel AF647. Image C: EBNA1 antibody from Abcam at channel AF488, showing no signal. Image D: Overlay of A, B, and C, confirming no signal bleed-through when using the EBNA1 antibody from Abcam. EBV Detection Using Invitrogen’s EBNA1 Antibody: Image E: DAPI staining highlighting the nuclear boundary of KS cells. Image F: EBNA1 antibody from Invitrogen at channel AF647. Image G: EBNA1 antibody from Invitrogen at channel AF488, showing no signal. Image H: Overlay of E, F, and G, confirming no signal bleed-through when using the EBNA1 antibody from Invitrogen. Kaposi Sarcoma Herpes Virus (KSHV) Detection Using Rat Anti-LANA Antibody: Image I: DAPI staining highlighting the nuclear boundary of KS cells. Image J: Rat anti-LANA antibody at channel AF488. Image K: Rat anti-LANA antibody at channel AF647, showing no signal. Image L: Overlay of I, J, and K, confirming no signal bleed-through when using Rat anti-LANA antibody. Note: All images were captured at 40X magnification with a KEYENCE microscope and analyzed using the KEYENCE BZ-X800 Analyzer. For journal presentation, images were cropped to a 300x300 pixel resolution using Microsoft’s Paint 3D software.
