# Supplementary figures and images for: Co-infection and co-localization of Kaposi sarcoma-associated herpesvirus and Epstein-Barr virus in HIV-associated Kaposi sarcoma: a case report
Source: Front Cell Infect Microbiol. 2023 Oct 20;13:1270935. doi: 10.3389/fcimb.2023.1270935 (PMC10623342; doi:10.3389/fcimb.2023.1270935)

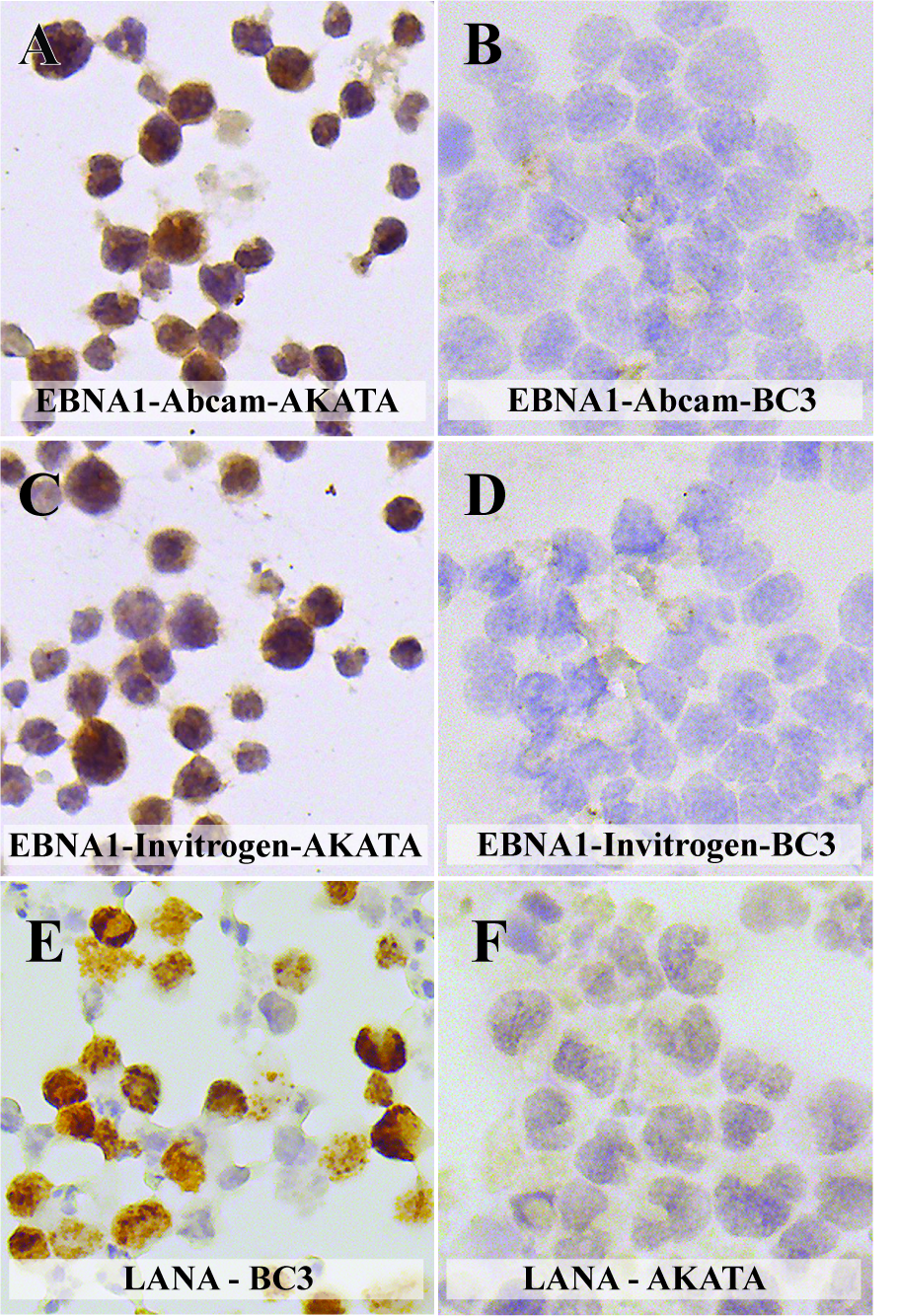

Supplement: Supplementary Figure 1 — Demonstrating Antibody Specificity through Immunohistochemistry (IHC). Given the potential sequence similarities between EBV and KSHV, both double-stranded DNA gamma herpesviruses, we conducted tests on Akata, Akata31, and BC3 cell lines to ensure no cross-reactivity of our antibodies. Image A: Akata Cell Line (EBV Infected): This image shows the cells stained with Epstein Barr virus’s nuclear antigen 1 (EBNA1) from Abcam. The positive staining confirms the presence of EBV infection. Image B: BC3 Cell Line: The cells were stained with EBNA1 from Abcam. The negative staining result confirms no cross-reactivity of EBNA1 with KSHV-infected BC3 cells. Image C: Akata Cell Line (EBV Infected): This image shows the cells stained with EBNA1 from Invitrogen. The positive staining indicates EBV infection. Image D: BC3 Cell Line: The cells were stained with EBNA1 from Invitrogen. The negative result confirms the absence of EBNA1 cross-reactivity with KSHV-infected BC3 cells. Image E: BC3 Cell Line (KSHV Infected): This image shows the cells stained with Kaposi sarcoma-associated herpesvirus-encoded latency-associated nuclear antigen (LANA) protein. The positive staining confirms KSHV infection. Image F: Akata Cell Line (EBV Infected): The cells in this image were stained with LANA. The negative staining result confirms no cross-reactivity of LANA with EBV-infected Akata cells. Note: All digital microscopic images of the stained slides were captured at X80 magnification using the MoticEasyScan Pro 6 scanner (Motic, USA) and analyzed using the Motic DSAssistant VM 3.0 software. For the journal presentation, images were cropped to a 300x300 pixel resolution using Microsoft’s Paint 3D software. [file Image_1.tif]

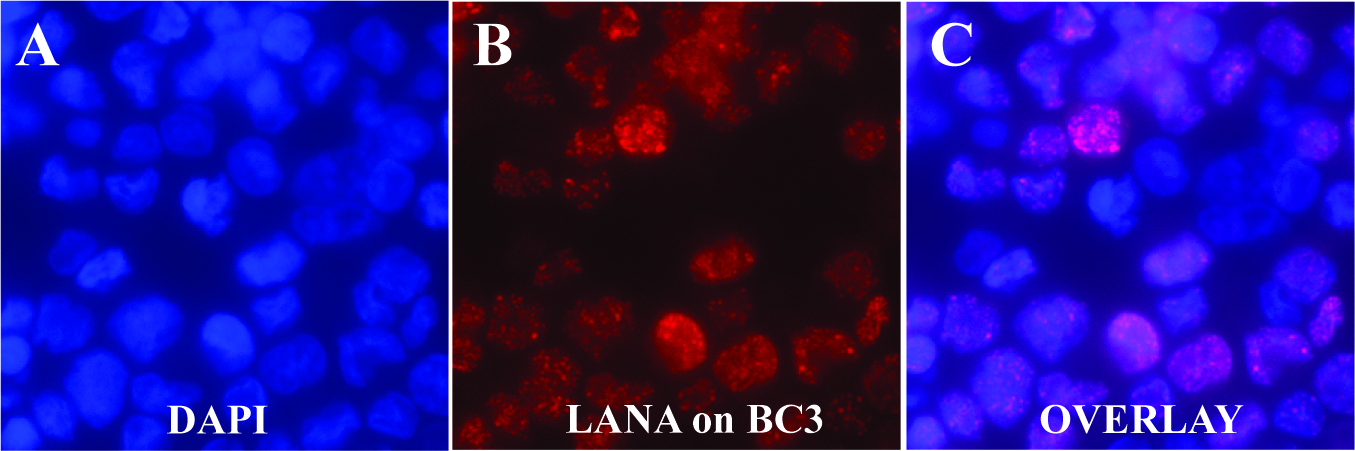

Supplement: Supplementary Figure 2 — Representative Immunofluorescence Assay Images of BC3 Cell Lines at 40X Objective Lens: BC3 cell lines serve as positive controls for LANA staining to detect Kaposi Sarcoma Herpes Virus (KSHV) infection. Image A: DAPI stain highlighting the nuclear boundary of BC3 cells. Image B: Detection of KSHV using the LANA Rat monoclonal antibody [LN53] (Ab4103). Image C: Overlay of DAPI and LANA (from Images A & B), confirming KSHV presence in the cell nucleus. Note: All digital images were captured at 40X magnification with a KEYENCE microscope and analyzed using the KEYENCE BZ-X800 Analyzer. For journal presentation, images were cropped to a 300x300 pixel resolution using Microsoft’s Paint 3D software. [file Image_2.tif]

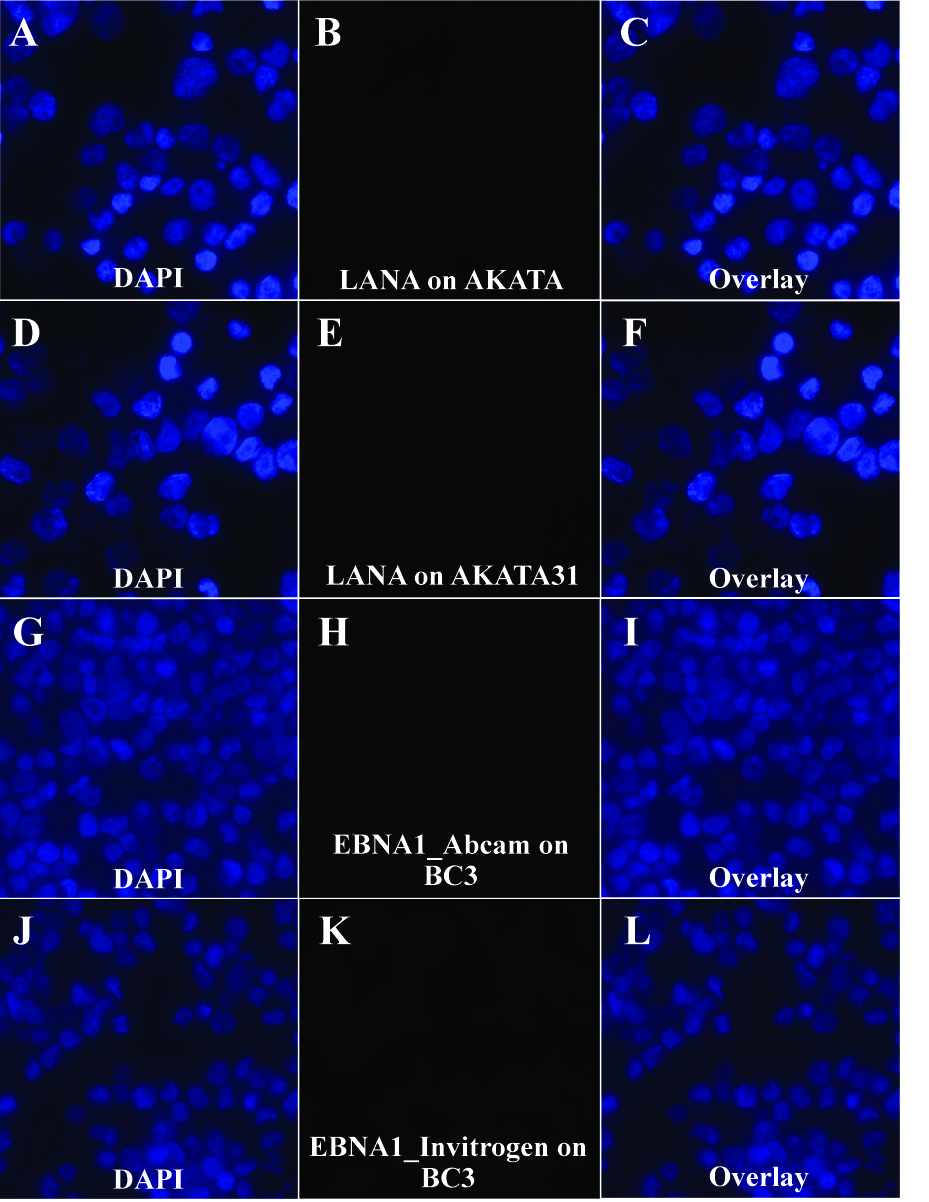

Supplement: Supplementary Figure 3 — Representative Immunofluorescence Assay Images using 40X Objective Lens: Demonstrating Antibody Specificity. Akata Cell Line (EBV Infected) images A-D). Image A: DAPI staining. Image B: Negative staining using Abcam’s Rat anti-LANA antibody, confirming LANA’s absence of cross-reactivity with EBV-infected cells. Image C: Overlay of A and B, emphasizing no cross-reactivity between LANA and EBV. Akata31 Cell Line (Non-EBV Infected) images D-F. Image D: DAPI staining. Image E: Negative staining using Abcam’s primary Rat anti-LANA, confirming LANA’s absence of cross-reactivity with Akata31. Image F: Overlay of D and E, emphasizing no cross-reactivity between LANA and the non-EBV-infected cell line. BC3 Cell Line (KSHV Infected) images G-I): Image G: DAPI staining highlighting the nuclear boundary. Image H: Negative staining using the EBNA1 antibody from Abcam, confirming EBNA1’s absence of cross-reactivity with KSHV-infected cells. Image I: Overlay of G and H, emphasizing no cross-reactivity between ABCAM’s EBNA1 and KSHV LANA. Image J: DAPI. Image K: Negative staining using the EBNA1 antibody from Invitrogen, confirming EBNA1’s absence of cross-reactivity with KSHV-infected cells. Image L: Overlay of J and K, emphasizing no cross-reactivity between Invitrogen’s EBNA1 and KSHV LANA. Note: All images were captured at 40X magnification with a KEYENCE microscope and analyzed using the KEYENCE BZ-X800 Analyzer. For journal presentation, images were cropped to a 300x300 pixel. [file Image_3.tif]

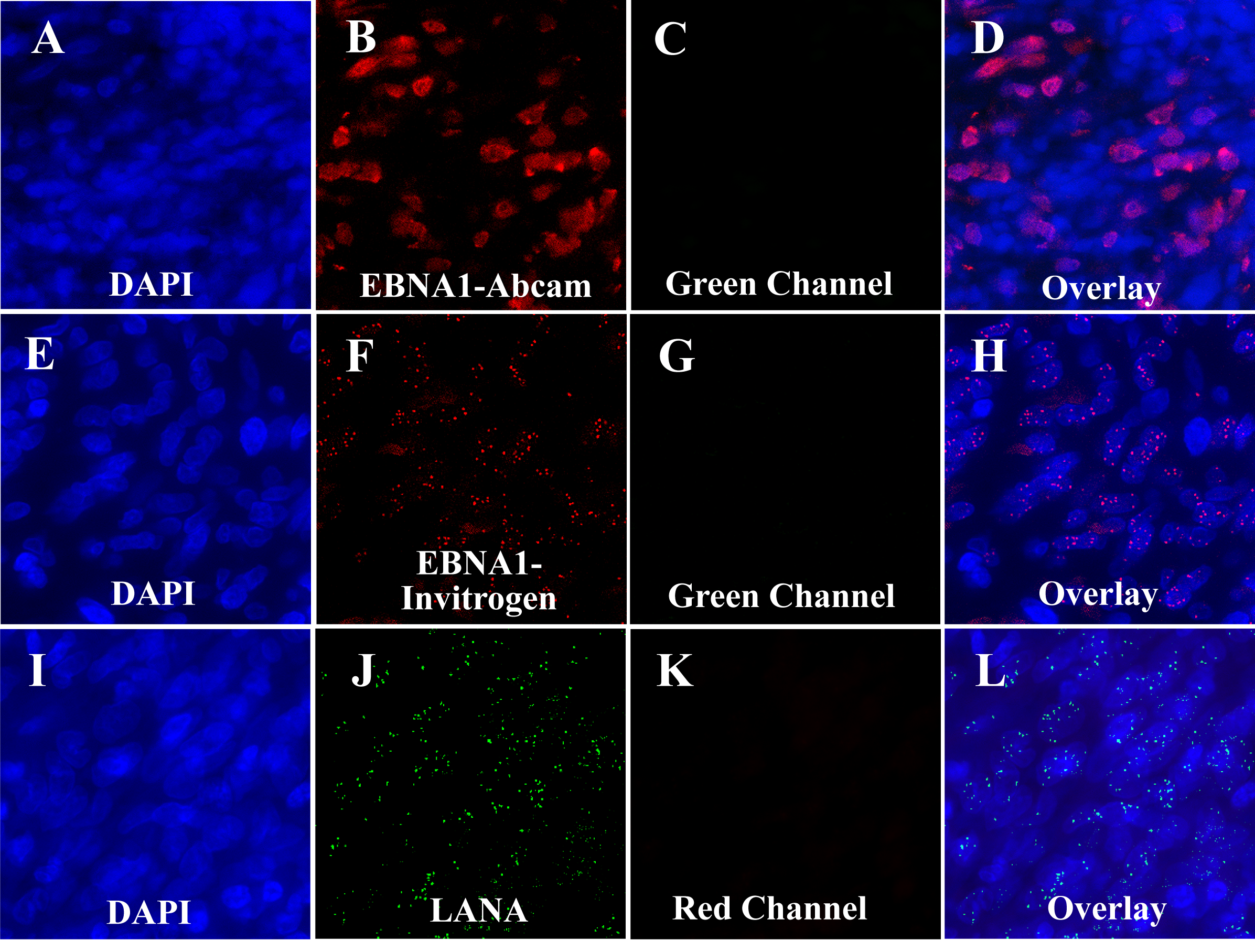

Supplement: Supplementary Figure 4 — Representative Immunofluorescence Assay Images at 40X Objective Lens: Ensuring No Signal Bleeding Across Color Channels. To ensure no “bleed through” signal from LANA and EBNA1 potentially confounding results, we stained slides exclusively with either anti-EBNA1 alone or anti-LANA alone, followed by scanning the slide at channels used for both EBNA1 (AF647, red color) and LANA (AF488, green color). Both color channels were displayed separately to verify that signals from one color were not bleeding into the other channel. EBV Detection Using Abcam’s EBNA1 Antibody: Image A: DAPI staining highlighting the nuclear boundary of the Kaposi sarcoma (KS) cells. Image B: EBNA1 antibody from Abcam at channel AF647. Image C: EBNA1 antibody from Abcam at channel AF488, showing no signal. Image D: Overlay of A, B, and C, confirming no signal bleed-through when using the EBNA1 antibody from Abcam. EBV Detection Using Invitrogen’s EBNA1 Antibody: Image E: DAPI staining highlighting the nuclear boundary of KS cells. Image F: EBNA1 antibody from Invitrogen at channel AF647. Image G: EBNA1 antibody from Invitrogen at channel AF488, showing no signal. Image H: Overlay of E, F, and G, confirming no signal bleed-through when using the EBNA1 antibody from Invitrogen. Kaposi Sarcoma Herpes Virus (KSHV) Detection Using Rat Anti-LANA Antibody: Image I: DAPI staining highlighting the nuclear boundary of KS cells. Image J: Rat anti-LANA antibody at channel AF488. Image K: Rat anti-LANA antibody at channel AF647, showing no signal. Image L: Overlay of I, J, and K, confirming no signal bleed-through when using Rat anti-LANA antibody. Note: All images were captured at 40X magnification with a KEYENCE microscope and analyzed using the KEYENCE BZ-X800 Analyzer. For journal presentation, images were cropped to a 300x300 pixel resolution using Microsoft’s Paint 3D software. [file Image_4.tif]
